# ZIF-8 with encapsulated dihydroartemisinin in a drug delivery system for protection against *Plasmodium berghei* ANKA-induced experimental cerebral malaria in C57BL/6N mice

**DOI:** 10.1128/spectrum.02366-24

**Published:** 2025-06-11

**Authors:** Yuting Li, Liu-Gen Li, Huiyin Zhu, Daiqian Zhu, Haimei Shi, Wei Wang, Jinyu Mo, Jianhai Yin, Tong-fei Li, Jian Li

**Affiliations:** 1 School of Basic Medical Sciences, Hubei University of Medicine 821843, Shiyan, Hubei, China; 2 Key Laboratory of National Health Commission on Parasitic Disease Prevention and Control, Jiangsu Provincial Key Laboratory on Parasites and Vector Control Technology, Jiangsu Institute of Parasitic Diseases 608817, Wuxi, Jiangsu, China; 3 National Institute of Parasitic Diseases, Chinese Center for Disease Control and Prevention (Chinese Center for Tropical Diseases Research), NHC Key Laboratory of Parasite and Vector Biology, WHO Collaborating Centre for Tropical Diseases, National Center for International Research on Tropical Diseases, Shanghai, China; National Institutes of Health, Rockville, Maryland, USA

**Keywords:** cerebral malaria, dihydroartemisinin, nanodrug, drug delivery, zeolitic imidazolate framework-8

## Abstract

Cerebral malaria (CM), primarily caused by *Plasmodium falciparum*, is the primary cause of malaria-related fatalities. CM treatment faces significant challenges due to limited therapeutic options and the emergence of antimalarial drug resistance. Dihydroartemisinin (DHA) is the first-line therapeutic agent for malaria. However, it encounters limitations such as poor solubility, inadequate selectivity, and rapid elimination. Here, we introduced a facile and effective approach using zeolite imidazolium framework-8 (ZIF-8) encapsulated with DHA (DHA@ZIF-8) and assessed its therapeutic efficacy in an experimental cerebral malaria model. The DHA@ZIF-8 demonstrated excellent drug-loading capacity, high stability, prolonged drug release, and improved targeted elimination of *Plasmodium* parasites within infected red blood cells (iRBCs). This minimized damage to brain microvascular endothelial cells (BMECs) and protected organs from injury, increasing the survival time of the infected mice. Compared to free DHA, DHA@ZIF-8 exhibited better antimalarial efficacy and almost no side effects. This study highlights ZIF-8’s potential as a reliable, stable, and efficient drug delivery vector for DHA to improve CM therapy.

**IMPORTANCE:**

For the treatment of human malaria, artemisinin-based drugs remain the first-line treatment option. However, their utility is constrained by their short half-life *in vivo*. Consequently, extending the duration for drug efficacy in the body is a critical issue that needs to be addressed. Metal-organic frameworks are a promising choice for drug loading. In the present study, DHA@ZIF-8 and DHA@MOF were constructed and characterized and were assessed in an experimental cerebral malaria model of C57BL/6 N mice induced by *Plasmodium berghei* ANKA strain. Data show that DHA@ZIF-8 has a worthy therapeutic effect on experimental cerebral malaria. It will offer a new option for human cerebral malaria (HCM) treatment.

## INTRODUCTION

Cerebral malaria (CM), primarily caused by *Plasmodium falciparum*, is associated with severe neurological complications and accounts for the majority of malaria fatalities (1). Even survivors also experience serious neurological consequences (2). Furthermore, the increasing spread of antimalarial drug resistance and insufficient therapeutic selectivity present significant challenges to effective malaria treatment (3, 4). Thus, the research and development of novel and effective drug formulations for malaria therapy is urgently needed (5). Dihydroartemisinin (DHA), with superior antimalarial efficacy compared to artemisinin (ART), remains the primary therapeutic option for CM. However, its application is limited by poor water solubility, a short circulating half-life, and nonspecific distribution (6). The integration of DHA with a nanocarrier represents a viable strategy to address these shortcomings (7, 8). Although some nanoparticles (NPs) have been used to deliver DHA, their clinical application is limited by several factors. These include the complexity and time-consuming nature of their production, insufficient drug-loading capacity, and uncontrollable drug release (9–12). Consequently, there is an urgent need for a safe platform that facilitates the effective carriage of DHA.

Metal-organic frameworks (MOFs), comprising organometallic clusters and linkers, have received significant attention (13). When downsized to the nanoscale as nanoscale MOFs (NMOFs), they become effective delivery vehicles, thanks to their nanostructure dimensions and substantial drug-carrying ability (14). As one of the most widely used NMOFs, zeolitic imidazolate framework-8 (ZIF-8), which is synthesized through the coordination of Zn^2+^ and 2-methylimidazole, has demonstrated significant promising drug delivery capabilities (15). ZIF-8 is characterized by remarkable heat stabilization and sustained drug release together with targeted drug delivery. Moreover, they maintain stability under physiological conditions while undergoing decomposition in environments characterized by low pH conditions (16). Hence, the reasonable design of a productive and uncomplicated drug package vehicle on the basis of ZIF-8 is highly important for addressing the challenges associated with the application of DHA.

In the present study, ZIF-8 loaded with the antimalarial drug DHA (DHA@ZIF-8) was applied to treat CM. Meanwhile, the effects of DHA, DHA@ZIF-8, and DHA@MOF for CM treatment were also assessed at the behavioral and histopathological levels. This work provides novel perspectives for the development of advanced nanotargeted approaches for the treatment of parasitic diseases.

## MATERIALS AND METHODS

### Preparation and characterization of DHA@ZIF-8

The synthesis of DHA@ZIF-8 was performed according to previously reported methods (17). DHA@ZIF-8 was obtained and maintained at 4°C for subsequent use. The morphological characteristics of ZIF-8 and DHA@ZIF-8 were examined by scanning electron microscopy (SEM) and transmission electron microscopy (TEM). The dimension and zeta potential of DHA@ZIF-8 were evaluated using a Malvern laser particle size analyzer.

### *In vitro* hemolysis analysis

For the hemolysis analysis (18), ZIF-8 and DHA@ZIF-8 were used at high (CZIF-8 = 0.34 mg/mL; CDHA@ZIF-8 = 0.54 mg/mL), middle (CZIF-8 = 0.17 mg/mL; CDHA@ZIF-8 = 0.27 mg/mL); and low (CZIF-8 = 0.017 mg/mL, CDHA@ZIF-8 = 0.027 mg/mL) concentrations. Blood from healthy mice was collected, subjected to centrifugation under conditions of 3,000 rpm for 15 minutes, and washed three times with saline to obtain red blood cells (RBCs). The 50% hematocrit (Hct) was obtained by adding normal saline to the cells. Normal saline (570 µL) with a 30 µL cell suspension was used as a negative control (without hemolysis). The positive control (100% hemolysis) was 30 µL of cells diluted with 570 µL of ultrapure water, which was used to lyse RBCs in hypotonic medium. Various concentrations of NPs were then added to the cell suspension, and each experimental condition (each ZIF-8 and DHA@ZIF-8 treatment) was performed in triplicate wells. The mixtures were subjected to incubation at 37°C for 1 h in a shaker. Next, the mixtures were centrifuged at 1,500 rpm for 10 min, after which the absorbance at 540 nm was quantified using a microplate reader (Gene Company Limited, Synergy HT, USA). A hemolysis ratio (A%) of less than 5% was considered nontoxic. The percentage of hemolysis was determined using the following equation:


$$\text{ Hemolysis }(\%)=\frac{A_{\text{test }}-A_{\text{neg }}}{A_{\text{pos }}-A_{\text{neg }}}\times 100$$

where A_test_ represents the absorbance of the ZIF-8 or DHA@ZIF-8 group, A_pos_ represents the absorbance of the group with 100% hemolysis, and A_neg_ means the absorbance of the group without hemolysis.

### Mice infected with *Plasmodium berghei* ANKA (PbA)

Six-week-old female C57BL/6 N mice were procured from LNCS Co., Ltd. (Changsheng, China) and maintained in specific pathogen-free environments. All the mice were acclimatized for 7 days prior to the experiment and were allowed access to a diet illuminated by ultraviolet light and purified water at room temperature and 60% relative humidity during the experiments. *Plasmodium berghei* ANKA strains were quickly thawed at 37°C. The persistence of parasitemia was achieved through continuous infection of the mice. The initiation of infection was triggered by the intraperitoneal injection of 1 × 10^6^ infected RBCs (iRBCs) (19).

### Experimental groups and drug administration

**Fig 1:**
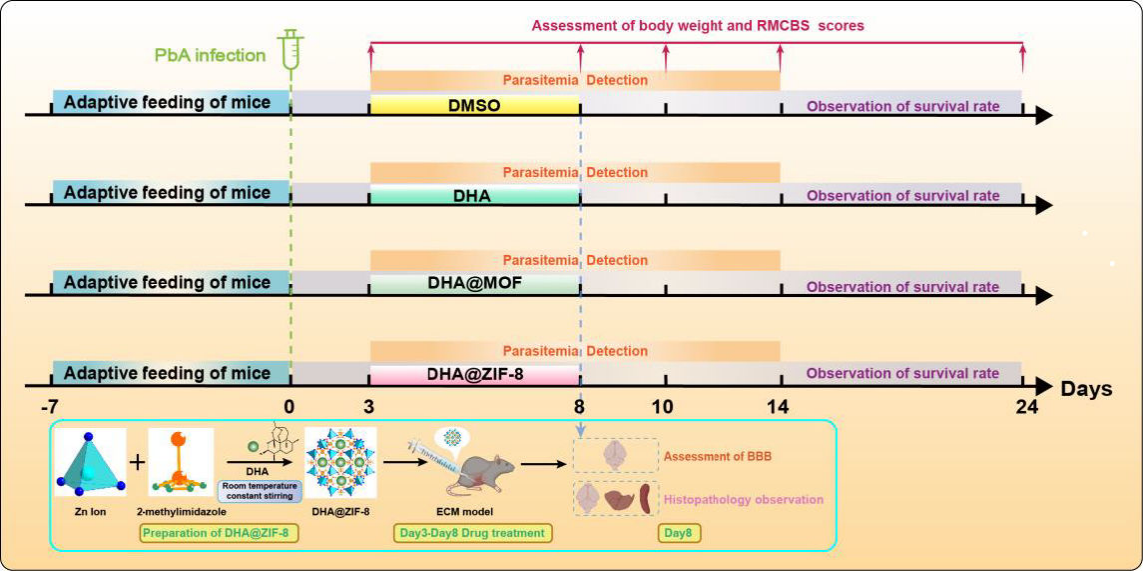
DHA@ZIF-8 was utilized for CM treatment and experimental grouping. Crystal structure of ZIF-8 and the preparation of DHA@ZIF-8 NPs and their application for antimalarial therapy. The orange and blue dots represent carbon and nitrogen atoms, respectively. The blue polygon represents a zinc ion. The day of infection was recorded as 0 dpi. The experimental cerebral malaria (ECM) model was established by infecting C57BL/6 mice with *Plasmodium berghei* ANKA (PbA) at 0 dpi. Therapy with dihydroartemisinin (DHA), DHA@MOF, and DHA@ZIF was administered at 3 dpi, with the respective administration times for each group shown. Mice were monitored for body weight, rapid murine coma and behavioral scale (RMCBS) score, parasitemia, survival rate, blood-brain barrier (BBB) integrity, and histopathology.

As demonstrated in Fig. 1, mice were randomly assigned to four groups: infection without drug treatment (PbA), DHA treatment (DHA), DHA@MOF treatment (DHA@MOF), and DHA@ZIF-8 treatment (DHA@ZIF-8). Three days after infection (day post infection, dpi), except for those in the PbA group, the mice in the groups of DHA, DHA@MOF, and DHA@ZIF-8 were treated with drugs for 5 consecutive days. The DHA, DHA@MOF, and DHA@ZIF-8 were solubilized in solutions comprising 5% dimethyl sulfoxide (DMSO) and normal saline based on a dosage of 3 mg/kg body weight. The mice in the PbA group were intraperitoneally administered 5% DMSO at 3 dpi to serve as a control. The day of infection was designated 0 dpi.

### Detection of basic indicators

The body weights, neurologic scores, parasitemia, and survival rates of the mice were recorded every day (Fig. 1). Neurological indicators were evaluated using the rapid murine Coma and Behavioral Scale (RMCBS) beginning at 0 dpi (20). The assessment is capable of evaluating a subject mouse within a few minutes (20). Thin blood smears of tail blood from mice were prepared and stained with Giemsa stains to assess parasitemia, which was quantified by counting the number of iRBCs with no less than 1,000 RBCs (21). The survival rate was recorded daily.

### Evaluation of blood-brain barrier (BBB) penetrability

Evans blue (EB) was prepared by diluting it in normal saline. A volume of 200 µL of 1% EB solution was administered to each mouse through the tail vein at 8 dpi. After 30 minutes of staining circulation, the mice were euthanized to gain the brain tissues. The tissues were promptly extracted and underwent incubation in 1 mL of formamide per sample at 37°C for 48 h. These compounds underwent centrifugation at 1,000 × *g* for 10 min. The absorbance at 630 nm was assessed via a microplate reader to quantify the EB concentration (18).

### Histopathology

These tissues of mice, each measuring 4 mm in thickness, were promptly harvested after euthanasia, fixed, and stained with hematoxylin-eosin (H&E) to assess microvascular destruction. Additionally, the quantification of hemozoin together with the regions of the red pulp (RePu) and white pulp (WhPu), measured using ImageJ software (version 10.0, USA), was done to evaluate the protection of the liver and spleen. Ten fields were randomly selected for each section (22), and images were acquired using Olympus cellSens (version 1.12, Japanese).

### Statistical analysis

Data were analyzed using the Statistical Package for Social Sciences (SPSS Inc., USA). Results were presented as means ± standard deviations (SDs). Survival analysis was performed by the log-rank (Mantel-Cox) test. Graphical representations were created via GraphPad Prism software (version 10.0, USA) and Origin 9.90. A *P* value of less than 0.05 was considered statistically significant. The sign symbols *, **, and *** indicate *P* < 0.05, *P* < 0.01, and *P* < 0.001, respectively. The *P* value less than 0.0001 is indicated by *P* < 0.0001.

## RESULTS

### Characterization of the DHA@ZIF-8

**Fig 2:**
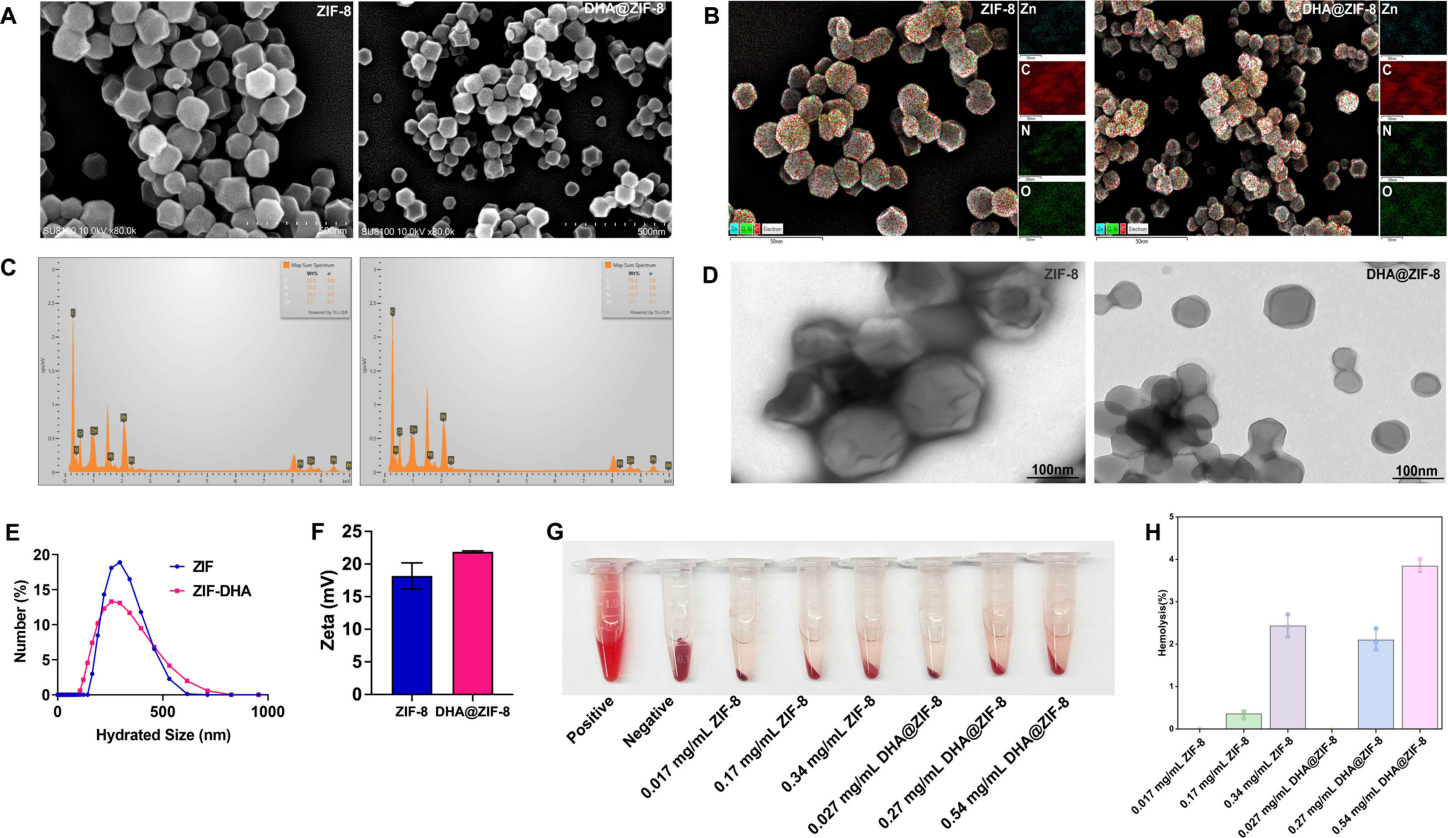
Characterization of DHA@ZIF-8. (**A**) SEM image of the successfully prepared ZIF-8 and DHA@ZIF-8. The particle size was approximately 100–150 nm. (**B**) SEM micrographs and EDX elemental mapping (Zn, C, N, and O) images of ZIF-8 and the as-synthesized DHA@ZIF-8. (**C**) Map of the sum of the spectra of ZIF-8 and DHA@ZIF-8. (**D**) TEM images of ZIF-8 and DHA@ZIF-8. Scale bars: 100 nm. (**E**) The hydrodynamic size of both ZIF-8 and DHA@ZIF-8 was approximately 250 nm, as characterized by a Malvern laser particle size analyzer. (**F**) Zeta potential results of the prepared ZIF-8 and DHA@ZIF-8. (**G**) Hemolysis observation of ZIF-8 and DHA@ZIF-8. (**H**) Hemolysis analysis of low (CZIF-8 = 0.017 mg/mL), middle (CZIF-8 = 0.17 mg/mL), and high (CZIF-8 = 0.34 mg/mL) concentrations of ZIF-8 and low (CDHA@ZIF-8 =0.027 mg/mL), middle (CDHA@ZIF-8 = 0.27 mg/mL), and high (CDHA@ZIF-8 = 0.54 mg/mL) concentrations of DHA@ZIF-8 incubated with RBCs.

DHA@ZIF-8 exhibited a characteristic crystal structure with dimensions ranging from 100 to 150 nm, as evidenced using SEM (Fig. 2A) and TEM (Fig. 2D). The elemental compositions of ZIF-8 and DHA@ZIF-8 are shown in Fig. 2B and C, respectively. The mean diameters of ZIF-8 and DHA@ZIF-8 were approximately 80 nm and 140 nm, respectively, indicating favorable stability (Fig. 2E). Furthermore, the zeta potential of DHA@ZIF-8 revealed a positive value and was verified using a Malvern zeta potential meter (Fig. 2F). Notably, this value slightly increased following the loading of DHA. Collectively, these findings strongly indicate the successful synthesis of DHA@ZIF-8.

### *In vitro* hemolysis analysis

In Fig. 2G, both low concentrations of ZIF-8 and DHA@ZIF-8 presented minimal hemolytic activity, as evidenced by the lighter supernatants in the ZIF-8 low-concentration (CZIF-8 = 0.017 mg/mL) group and the DHA@ZIF-8 low-concentration (CDHA@ZIF-8 = 0.027 mg/mL) group. The hemolysis rates of the supernatants in the ZIF-8 middle-concentration (CZIF-8 = 0.17 mg/mL) group and DHA@ZIF-8 middle-concentration (CDHA@ZIF-8 = 0.27 mg/mL) group were 0.36% and 2.10%, respectively (Fig. 2H). As the drug concentration increased, the supernatant started to turn red. Quantitative analysis showed that hemolysis rates increased to 2.43% and 3.84% for high-concentration ZIF-8 (0.34 mg/mL) and DHA@ZIF-8 (0.54 mg/mL), respectively, which remain within the safe range (Fig. 2H). RBCs in the positive control tube completely ruptured, leading to the release of hemoglobin into the supernatant. No hemolytic activity was observed in the supernatant, and intact red blood cells aggregated to form a dense dark pellet at the bottom of the negative control. Consequently, this drug delivery platform could serve as an effective vehicle for mitigating the harmful effects and drug tolerance linked to DHA.

### Body weight and neurological signs

**Fig 3:**
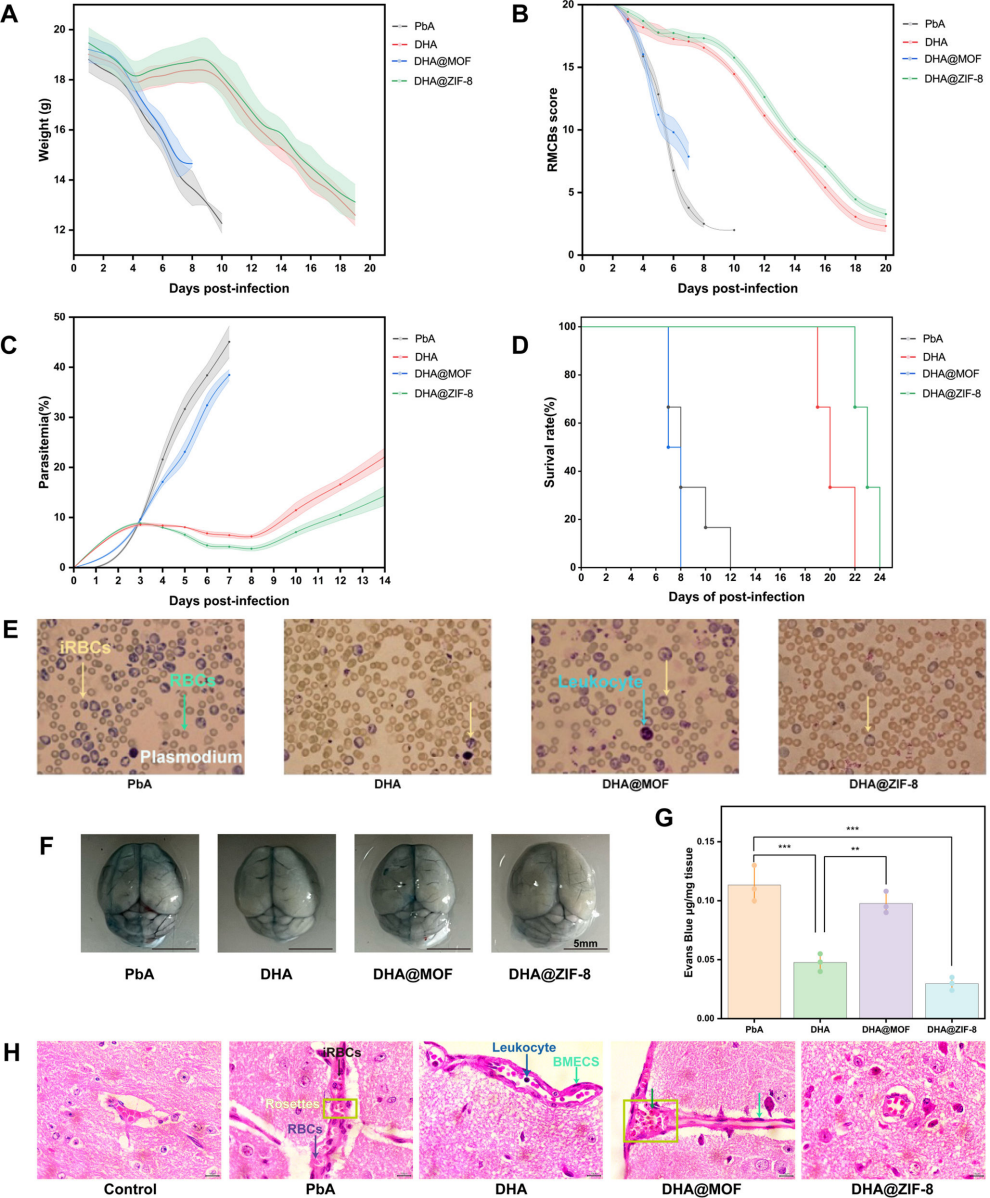
Histopathological staining of the brain, liver, and spleen after different treatments. (**A**) Body weights of the ECM-treated mice receiving different treatments. (**B**) RMCBS score curve of ECM model mice receiving different treatments. (**C**) Parasitemia in ECM-treated mice receiving different treatments. (**D**) Survival rate curve analysis of ECM-treated mice. Survival rate analysis was assessed by the log-rank (Mantel-Cox) test. (**E**) Representative images of parasitemia following various treatments. (**F**) Representative images of brain tissue following various treatments. (**G**) Evaluation of vascular leakage via the Evans blue test to assess BBB permeability. (**H**) Hematoxylin-eosin (H&E) staining of the brain (magnification, ×1,000). The yellow arrow points to red blood cells (RBCs). The red arrow points to brain microvascular endothelial cells (BMECs). Scale bars: 10 µm.

With the exception of the PbA and DHA@MOF groups, the mice in the DHA and DHA@ZIF-8 groups presented an initial decrease in body weight, which was then followed by an increase and a subsequent decline from 0 dpi onward (Fig. 3A). At 8 dpi, the mean weight of the PbA group decreased to less than 13 g. The mean body weight of the DHA@MOF group significantly exceeded that of the PbA group (*P* = 0.003). At 18 dpi, the DHA@ZIF-8 group mice presented higher weight than the DHA group mice (*P* = 0.047).

All groups of mice presented a decreasing trend in RMCBS scores (Fig. 3B). The PbA group began to exhibit signals of diminished exploratory behavior, reduced limb strength, and impaired gait stability at 4 dpi, accompanied by a rapid decline in RMCBS scores. At 10 dpi, the RMCBS score reached a value of 1.8. The DHA@MOF treatment resulted in a rapid decline in RMCBS levels, similar to the PbA group. In comparison, the other two treatments presented significantly higher RMCBS scores during the same period. The RMCBS scores of DHA and DHA@ZIF-8 groups slightly decreased, but the RMCBS scores remained within the normal range from 4 dpi to 8 dpi. The RMCBS scores of the DHA and DHA@ZIF-8 groups remained stable until experiencing a delayed decrease. At 8 dpi, RMCBS scores of the mice in the DHA@MOF group were significantly higher than that of the PbA group (*P* = 0.007). The highest RMCBS scores were assessed in the DHA@ZIF-8 group. Compared with the DHA group, the DHA@ZIF-8 group exhibited a higher score (*P* = 0.019).

### Parasitemia and survival rates

Fluctuations in parasitemia over the 14-day infection period were observed. As shown in Fig. 3C and E, measurements of peripheral parasitemia indicated an increase in parasitemia levels across all groups during the initial 3 dpi. Following the initiation of antimalarial drug therapy, a reduction in parasitemia was observed in both the DHA and DHA@ZIF-8 treatment groups. Compared with the DHA@MOF (*P* = 0.001) and DHA (*P* = 0.002) groups, the DHA@ZIF-8 group presented obviously lower parasitemia on day 8 post-infection. A significant difference between the PbA and MOF@DHA groups was also observed (*P* = 0.027). The peripheral blood erythrocytes in the DHA@ZIF-8 group exhibited the most intact morphology with the fewest infected iRBCs could be observed after continuous administration of the drug for 5 days (on 8 dpi) (Fig. 3E), which indicates that the protective effect on erythrocytes was superior in the DHA@ZIF-8 group compared to the DHA group. In contrast, the erythrocyte morphology in the PbA group was the most severely compromised, with the highest number of iRBCs.

The DHA@ZIF-8 group exhibited the highest survival rate across all the experimental groups (Fig. 3D). The PbA mice began to die at 7 dpi and presented specific ECM symbols and high parasitemia. No mice exhibited mortality attributable to ECM prior to 19 days in the DHA and DHA@ZIF-8 groups. No statistical differences were observed in survival rates between the PbA group and the DHA@MOF group (*P* = 0.192), but a significant difference was found between the DHA@MOF and DHA@ZIF-8 groups (*P* < 0.0001) and between DHA@ZIF-8 and DHA groups (*P* = 0.003). These findings indicate that DHA@ZIF-8 has superior efficacy in the treatment of the ECM model compared with DHA.

### Protection on the brain

Visual assessment coupled with EB quantification revealed that there was evident leakage of EB in the PbA group. In contrast, the DHA-treated group exhibited a lesser degree of leakage. However, no visible leakage could be observed in the DHA@ZIF-8 group (Fig. 3F). During the therapeutic application, there was no statistical difference in terms of EB leakage between the DHA group and the DHA@ZIF-8 group (*P* = 0.21). In addition, the level of EB leakage in the DHA@MOF group was higher than that in the DHA@ZIF-8 group (*P* < 0.0001). Compared with PbA, DHA@MOF had a more pronounced effect on BBB permeability (*P* = 0.30) (Fig. 3G).

The DHA@ZIF-8 group demonstrated a significant reduction in the accumulation of leukocytes and a decrease in the isolation of iRBCs (Fig. 3H). The PbA group exhibited significant accumulation of iRBCs and inflammatory cells within the cerebral vasculature. This aggregation led to the development of rosette-like structures, accompanied by the emergence of scattered hemorrhagic lesions surrounding the blood vessels. The DHA group exhibited a limited presence of inflammatory cells, along with several dispersed hemorrhagic lesions in proximity to the vessels. In mice treated with DHA@ZIF-8, the capillaries exhibited a near absence of iRBCs and minimal inflammatory cell adhesion. Additionally, there was limited aggregation of leukocytes within the cerebral vessels. In contrast, mice treated with DHA@MOF presented greater deposition of leukocytes within the luminal space. These findings indicated that DHA@ZIF-8 enhanced cerebral vascularity and substantially protected against brain injury caused by the ECM.

### Protection on the liver

**Fig 4:**
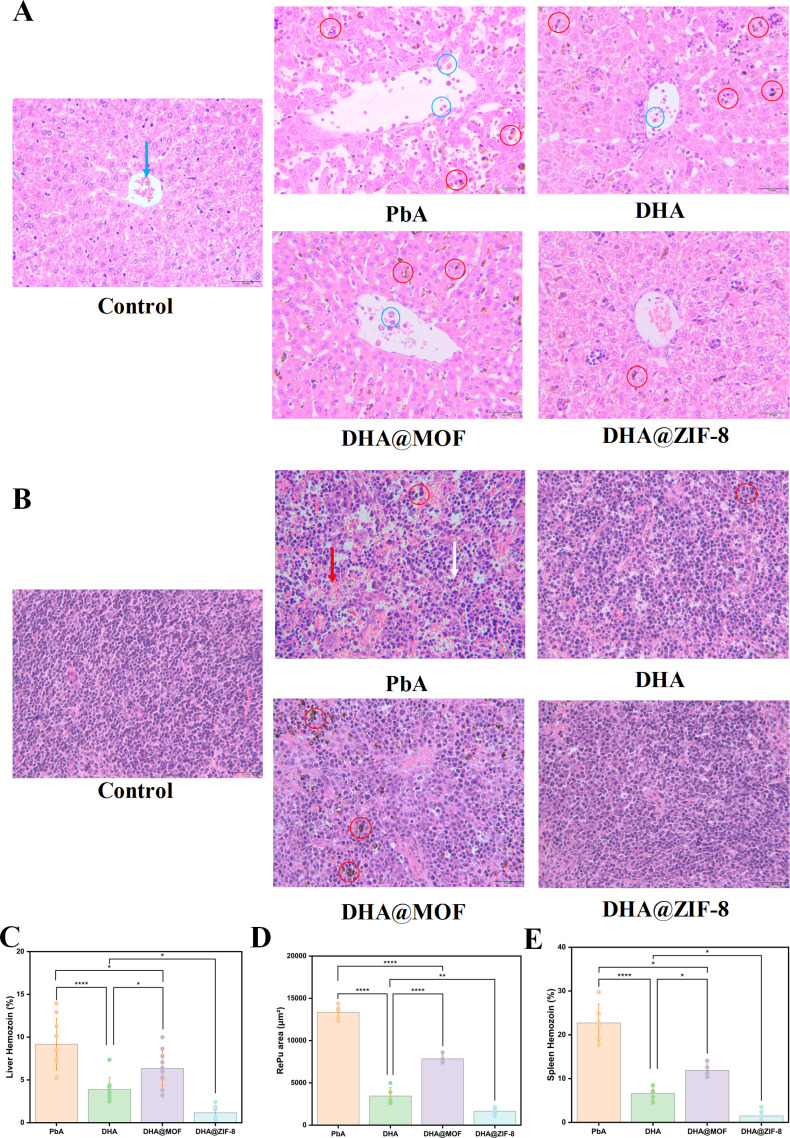
Monitoring of indicators associated with the ECM model receiving different treatments. (**A**) H&E staining of the liver (magnification, ×400). The blue arrow, blue circle, and red circle represent red blood cells, adherence, and hemozoin, respectively. Scale bars: 20 µm. (**B**) H&E staining of the spleen (magnification, ×400). The red arrow, white arrow, and red circle represent red pulp (RePu), white pulp (WhPu), and hemozoin, respectively. Scale bars: 20 µm. (**C**) Analysis of the proportions of hemozoin in the liver and spleen in the different treatment groups. (**D**) Quantification of the red pulp (RePu) area for the different treatment groups. (**E**) Analysis of the proportions of hemozoin in the spleen for the different treatment groups. The statistical analysis method was one-way ANOVA, and the least significant difference (LSD) test was used for each one-way ANOVA. The error bars indicate the standard deviation. Scale bars: 5 mm. *, *P* < 0.05; **, *P* < 0.01; ***, *P* < 0.001.

The DHA@ZIF-8 group had the most complete structure of liver lobules and exhibited the most significant protective effects on liver function among all the groups (Fig. 4A). The structure of the liver in the PbA group was significantly impaired, as characterized by darker dyeing of the hepatocyte cytoplasm. Moreover, the liver presented an abundance of lipid vacuoles. In addition, a significant accumulation of inflammatory cells was observed within the veins. Similarly, the DHA@MOF group exhibited serious structural damages to the hepatic lobules, accompanied by notable darkening of the liver structure. In the DHA group, the hepatic plates exhibited a loose arrangement, and the quantity of inflammatory cells obstructed the veins with a diminished deposition of hemozoin. In comparison to the DHA group, DHA@ZIF-8 exerted more evident protection on the liver, characterized by a more compact organization of hepatocytes and a notable decrease in inflammatory cell recruitment. In the DHA@ZIF-8 group, the hepatic cells of mice displayed a tight radial arrangement around the central vein, with well-defined cell boundaries.

Quantitative analysis was carried out to quantify hemozoin accumulation in the liver (Fig. 4C). The DHA@ZIF-8 group presented the lowest concentration of hemozoin. The efficacy of DHA in conjunction with ZIF-8 in promoting hemozoin metabolism was superior to that of DHA alone. Compared to the DHA group, a notable distinction was found in the DHA@ZIF-8 group (*Р* = 0.023). Additionally, a significant difference in hemozoin levels was observed between the DHA@MOF group and the PbA group (*Р* = 0.016). Therefore, DHA@ZIF-8 could serve as a potential therapeutic option for the CM treatment.

### Protection on the spleen

Histopathological assessment of the spleen in the PbA and DHA@MOF groups showed significant expansion of the red pulp (RePu) along with a reduction in white pulp (WhPu), indicative of severe anemia and increased extramedullary hematopoiesis (Fig. 4B). In the DHA group, the RePu was smaller than in the aforementioned two groups, suggesting a higher severity of anemia. In the other two groups, the RePu proportions were greater than in the DHA@ZIF-8 group. The spleen architecture of the DHA@ZIF-8 group closely resembled that of the control group. The RePu region in the PbA group was significantly larger than that in the DHA@MOF group (*P* < 0.0001). In comparison to the DHA group, the RePu with DHA@ZIF-8 exhibited a significant reduction (*Р* = 0.006), suggesting a reduction in extramedullary hematopoiesis. Compared with the PbA group, the DHA@MOF group tended to have a reduced RePu area (*P* < 0.0001). Although DHA@MOF decreased the RePu region, extramedullary hematopoiesis remained persistent. For DHA@ZIF-8, it decreased the RePu area and alleviated symptoms associated with anemia (Fig. 4D). Quantitative evaluation of splenic hemozoin revealed that DHA@ZIF-8 treatment resulted in the lowest hemozoin concentration (Fig. 4E). A notable distinction was observed between the DHA@ZIF-8 and the DHA groups (*P* = 0.013).

## DISCUSSION

Traditional therapies for HCM are limited by drug resistance and nonspecific targeting, which necessitates the administration of high-dose medications and consequently brings about associated toxicity (23). Fortunately, the integration of DHA with nanodrugs is likely to be a promising strategy to overcome these limitations (7, 8). In the present study, two metal nanoparticles of DHA@ZIF-8 and DHA@MOF were developed and evaluated for their therapeutic effectiveness in ECM. Compared with free DHA, DHA@ZIF-8 exhibited more accurate and targeted delivery. It also achieved higher drug encapsulation efficiency and exhibited a sustained release pattern. Most importantly, it displayed superior antimalarial effects, significantly improving the neurological, pathological, and behavioral outcomes in CM mice. Meanwhile, DHA@ZIF-8 significantly delayed the decrease in body weight and RMCBS score of mice, reduced the level of parasitemia, improved blood-brain barrier permeability, and pathological damage to the tissue caused by CM, prolonged the survival rate of the infected mice, and exhibited more efficacy compared with other therapeutic drugs.

ZIF-8 serves as an optimal carrier for drug delivery and sustained release (24), and DHA@ZIF-8 maintains stability under physiological conditions and degrades in acidic environments. In another study, DHA@ZIF-8 also demonstrated antitumor therapeutic efficacy by suppressing the generation of reactive oxygen species (ROS) and promoting the apoptosis of cells (17). Compared with free DHA, DHA@ZIF-8 improved antimalarial outcomes in the ECM. Owing to high Fe^2+^ concentrations, DHA@MOF exhibited toxicity and did not yield ideal consequences. However, the DHA@MOF group exhibited toxicity at high MOF concentrations, which could be attributed to oxidative stress induced by an overabundance of ROS, which will cause harm to proteins, lipids, and DNA within mitochondria, thereby fostering inflammation, apoptosis, or necrosis (25). The toxic properties of MOF may also result from its dissolution in aqueous solutions and the subsequent release of Fe^2+^ (26). This finding elucidates that even though DHA@MOF has potential applications in fields like tumor therapy, the DHA@MOF group in this study failed to achieve the desired results. Instead, it led to higher concentrations of Fe^2+^, which exacerbated organ damage in mice. For instance, the brain, liver, and spleen were affected, and the mortality rate of mice increased.

There are several limitations in the present study. For example, the precise mechanism by which DHA@ZIF-8 acts against ECM remains unclear. In future, we will further investigate the specific molecular protection mechanism associated with DHA@ZIF-8 for CM through RNA sequencing (RNA-seq), bioinformatics analysis, and multiomics platforms to identify new targets and evidence. Moreover, DHA encapsulated within biologically inspired membranes originating in microvascular endothelial cells of the brain was able to enhance the targeted killing effect on resident iRBCs according to our prior study (22). Thus, DHA@ZIF-8 NPs can be encapsulated in BMECs to target iRBCs in the blood and prevent damage to BMECs in the CM.

### Conclusions

In summary, we have successfully constructed and screened DHA@ZIF-8 NPs for drug loading. The findings reveal that, when compared to free DHA, DHA@ZIF-8 demonstrates superior antimalarial therapeutic outcomes. It highlights the potential application of DHA@ZIF-8 as a secure and constant framework to develop an extremely effective drug transportation platform for CM treatment, providing novel perspectives on the development of nanoparticle-based approaches for the therapy of HCM and infectious diseases.
